# Comparative analysis of the immunogenicity of monovalent and multivalent rotavirus immunogens

**DOI:** 10.1371/journal.pone.0172156

**Published:** 2017-02-16

**Authors:** Kai Mi, Xia Ou, Lili Guo, Jing Ye, Jinyuan Wu, Shan Yi, Xianglian Niu, Xiaoqin Sun, Hongjun Li, Maosheng Sun

**Affiliations:** 1 Yunnan Key Laboratory of Vaccine Research and Development on Severe Infectious Disease, Institute of Medical Biology, Chinese Academy of Medical Sciences & Peking Union Medical College, Kunming, Yunnan Province, the People’s Republic of China; 2 School of Basic Medicine, Kunming Medical University, Kunming, Yunnan Province, the People’s Republic of China; University of Liverpool, UNITED KINGDOM

## Abstract

The strategies for developing rotavirus (RV) vaccines have always been controversial. At present, both the monovalent RV vaccine and the multivalent RV vaccine have displayed excellent safety and efficacy against RV infection and shown cross-reactive immunity, which laid the question whether the multivalent RV vaccine could be replaced by the monovalent RV vaccine. In this study, we focused on comparing the immunogenicity (serum neutralization activity and protection against homotypic and heterotypic RVs’ challenge) of individual standard RV strains (monovalent RV immunogens) and different combinations of them (multivalent RV immunogens). In result, RV immunogens showed general immunogenicity and heterotypic reaction but the multivalent RV immunogens exhibited greater serum neutralization activity and stronger heterotypic reaction than the monovalent RV immunogens (P<0.05). As to the protection, the multivalent RV immunogens also revealed more rapid and stronger protection against homotypic and heterotypic RVs’ challenge than the monovalent RV immunogens. The results demonstrated that both the monovalent and multivalent RV immunogens exhibited high immunogenicity, but the monovalent RV immunogens could not provide enough neutralization antibodies to protect MA104 cells against the infection with heterotypic RV strains and timely protection against homotypic and heterotypic RVs, so the multivalent RV vaccine could not be replaced by the monovalent RV vaccine.

## Introduction

Rotaviruses (RVs) are the predominant cause of severe dehydrating diarrhea among infants and young children worldwide. It has been estimated that 39% of these diarrhea deaths in children under 5 years of age are due to RVs [1]. This disease has resulted in the deaths of nearly 450,000 young children less than 5 years of age globally each year and has been regarded to place a heavy burden on both developed and developing countries [2–3]. To date, no specific drugs for the treatment of RV infections have been identified. As a consequence, the development of RV vaccines is the only effective strategy to prevent and control RV infection.

RVs are important members of the Reoviridae family of icosahedral viruses, which are composed of 2 to 3 protein layers of capsid that enclose a genome consisting of 10 to 12 segments of double-stranded RNA (dsRNA) [4]. RV is a unique type of virus that contains multiple G genotypes and P genotypes, which are determined based on the structural viral protein VP7 (a glycoprotein or G-type antigen) and VP4 (a protease-sensitive protein or P-type antigen), respectively. Both VP7 and VP4 protein induces neutralization antibody responses. At present, 27 G genotypes (G1-G27) and 35 P genotypes (P[1]-P[35]) have been described for RVs [5].

The diversity of the combination of G and P genotypes represents a massive barrier to the development of RV vaccines. Fortunately, the reassuring results of epidemiological investigation suggest that not all RVs are prevalent during RV seasons and that only five genotypes, G1P[8], G2P[4], G3P[8], G4P[8] and G9P[8] are responsible for the vast majority of diarrheal diseases caused by human RVs [6–7]. In 2006, two live attenuated RV vaccines were used to protect young children against severe RV-induced gastroenteritis [8]. RotaTeq, a pentavalent human-bovine reassortant vaccine comprising the human G genotypes G1, G2, G3, G4 and P genotype P[8] in a bovine WC3 RV strain background, and Rotarix, a monovalent human vaccine containing a G1P[8] RV strain, have been licensed in many countries to date. These developments could be regarded as a turning point in the battle against RVs after Rotashield was suspended by the United States Centers for Disease Control (US CDC) in 1999 because of its side-effect of intussusception, which was a major setback in RV vaccine development [9–10]. In China, Oral rotavirus (live) vaccine, a monovalent vaccine involving a G10P[12] RV strain (Lanzhou Lamb Rotavirus, LLR) which were produced by Lanzhou Institute of Biological Products, has been approved for human use in 2001 and the sale has been accumulated to more than 30 million doses on the market [11]. No inactivated RV vaccine has been licensed to date. All the live attenuated RV vaccines mentioned above (both monovalent and multivalent RV vaccines) have displayed excellent safety and efficacy against RV diseases [12–16]. Moreover, the recent trials have demonstrated that both the monovalent RV vaccines (Rotarix and the Chinese oral rotavirus (live) vaccine) and the multivalent RV vaccine (RotaTeq) were effective against severe gastroenteritis caused by diverse circulating RV types, including RVs carrying different G genotypes from the vaccine strains [11, 17–18], which laid the question whether the multivalent RV vaccine could be replaced by the monovalent RV vaccine if both of the monovalent and multivalent RV vaccines could induce heterotypic antibodies and cross protection. Therefore, the strategies used to develop RV vaccines have been still controversial.

In this study, we specifically examined three standard RV strains, Wa (G1P[8]), SA11 (G3P[1]), Gottfried (G4P[6]) which carried different G genotypes and P genotypes, to evaluate the serum neutralization activity of them (monovalent RV immunogens) and different combinations of them (multivalent RV immunogens) and the protection against homotypic and heterotypic RVs’ challenge to provide a theoretical basis for the future development of RV vaccines.

## Materials and methods

### Ethics statement

Experimental animals were provided and raised by the Institute of Medical Biology Animal Center according to institutional guidelines. This research was approved by the Ethics Committee of the Institute of Medical Biology (YISHENGLUNZI [2011] 16). Specific pathogen-free (SPF) female ICR mice (18–25 g, 60–90 days old, unmated) were used in this experiment. The mice were raised in a comfortable environment (room temperature: 25°C; humidity: 50%-60%). The food, drinking water and padding were supplemented and changed at regular intervals. We monitored the mice twice a day and check the health condition of the mice, including the appetite, the eye contact, the body hair, the vitality, the wound and the shape of faeces. The non-feeder and wild animals were forbidden to enter into the feeding zone to keep the mice healthy. During the experiments, we used pentobarbital sodium (2% 0.1ml) as anesthetic to minimize the animal suffering. After the experiments, the mice were euthanized by dislocation of cervical vertebra. There was no mortality prior to the end of the experiment.

### Preparation of the RV immunogens

MA104 cells (passage 28, from the Institute of Medical Biology, Chinese Academy of Medical Science & Peking Union Medical College), which are sensitive to RVs, were used as the cellular matrix to amplify the RVs. The standard RV strains Wa (G1P[8]), SA11 (G3P[1]) and Gottfried (G4P[6]) (stored at -80°C, from the “Master Seed” of the Department of Molecular Biology, Institute of Medical Biology, Chinese Academy of Medical Science & Peking Union Medical College) were prepared for the “Working Seed”. Prior to infection, the RVs were activated with 20 μg/ml trypsin for 45 min at 37°C. After being absorbed for 90 min at 37°C, the RVs were cultured for 48–72 h in serum-free MEM containing 1 μg/ml trypsin at 37°C. When a cytopathic effect (CPE) was observed in more than 80% of the MA104 cells, freeze-thaw (-20°C) of the viral culture media was repeated three times. Then, the cell debris was discarded via centrifugation at 8,000 rpm for 20 min at 4°C. The RV suspensions were concentrated using a Pellicon XL Ultrafiltration Module (Biomax 50 kDa 0.005 m^2^, Millipore, PXB050A50) and were then purified using a Q Sepharose Fast Flow anion exchanger system (GE Healthcare, 17-0510-01) [19]. Finally, the RV suspensions that were eluted in NaCl solution and then desalted using a Sephadex G-25 Superfine column (GE Healthcare, 17-0033-01). The purified RV suspensions were used as immunogens for the subsequent vaccination of the experimental animals.

### Measurement of the cell culture infective dose 50% (CCID_50_) of the RV immunogens

The quantities of RV immunogens were expressed as infectious titers, which were defined as the CCID_50_, and the immunofluorescence assay (IFA) was used as the primary reference method. The RV suspensions were diluted in serum-free MEM to a starting dilution of 10^−1^, and a series of seven 10-fold dilutions was performed to generate a dilution range from 10^−1^ to 10^−8^. The diluted RV suspensions were transferred to 96-well plates covered with a confluent monolayer of MA104 cells and were cultured for 16 h at 37°C. The MA104 cells that were infected with the RVs were fixed to the 96-well plates using an equal volume of pre-chilled formaldehyde solution for 0.5 h at 4°C. After the 96-well plates were air-dried, guinea pig serum samples against corresponding RV genotypes (stored at -20°C, prepared by the Department of Molecular Biology, Institute of Medical Biology, Chinese Academy of Medical Science & Peking Union Medical College) that were diluted to 1:500 (v/v) in phosphate buffered saline (PBS) containing 2% (m/v) bovine serum albumin (BSA, Biosharp, BS043D) were added to the 96-well plates. After incubation for 1 h at 37°C, fluorescein isothiocyanate (FITC)-conjugated goat anti-guinea pig IgG (entire molecule) (SIGMA, F6261), which was diluted to 1:2000 (v/v) in PBS, was added to the 96-well plates in the dark. After another incubation for 1 h at 37°C, the specific green fluorescence was observed using a fluorescence microscope. MA104 cells in serum-free MEM were used as a negative control. The sample in a given well was considered to be positive if one MA104 cell displaying green fluorescence was found in the well. The proportion of positive wells for each dilution, which contained 10 replicates, was represented as the mean percentage. The CCID_50_ was calculated using the Reed-Muench formula as follows:

proportionate distance=(percentagepositiveabove50%-50%) / (percentagepositiveabove50%-percentagepositivebelow50%);

$$lg\;CCID_{50}=lg\;\left(dilutionabove50\%\right)\;+\;proportionate\;distance\times lg\;\left(dilutionfactor\right).$$

### ELISA test for serum RV-specific antibodies

The wells of the ELISA plate were coated with a goat anti-rotavirus polyclonal antibody (Millipore, AB1129) diluted to 1:1000 (v/v) in carbonate-bicarbonate buffer (pH 9.6) overnight at 4°C. After washing three times with PBS containing 0.05% (v/v) Tween-20 (PBS-T), the ELISA plates were blocked with 2% (w/v) BSA (Biosharp, BS043D) diluted in PBS for 2 h at 37°C. Then, the diluted purified RV suspensions carrying different genotypes (100 μl/well) were incubated for 1 h at 37°C. The serum samples (50 μl/well) from the pre-immunized ICR mice were gradient-diluted (dilution range from 10- to 1280-fold in PBS) and incubated for 1 h at 37°C. After washing, the serum samples were incubated in horseradish peroxidase (HRP)-conjugated goat anti-mouse IgG (H+L) (Millipore, AP308P) diluted to 1:2000 (v/v) in PBS for 1 h at 37°C. Soluble TMB Substrate Solution (TIANGEN, PA107-01) was used to generate a colorimetric reaction, which was terminated via the addition of 2 mol/L H_2_SO_4_. The optical densities (ODs) at 450 nm were measured using a universal microplate reader (ELx 800, Bio-Tek, USA). The PBS was used as negative controls. A serum sample was determined to be positive if the OD value was greater than or equal to the average OD value of the negative control + 0.1. The serum RV-specific IgG levels were defined as the reciprocal of the highest dilution.

### Immunization of the experimental animals

Prior to immunization, the serums of all the ICR mice used in this experiment were detected via ELISAs to confirm that the ICR mice had not been infected with other RVs. Three doses (100 μl/dose) were administered every 15 days during the animal experiments via the oral route without adjuvant. Blood was collected from the caudal vein of the immunized ICR mice before immunization, 15 days after each immunization and 60 days after the latter booster immunization. Then, serum samples were obtained via centrifugation at 3,000 rpm for 30 min, incubation in a water bath at 56°C for 30 min, and incubation overnight at 4°C to prepare the samples for the subsequent evaluations.

### RV oral challenge

The female ICR mice in each group were mated with male ICR mice two weeks after the third dose. Thirty newborn mice were chosen from each group and divided into three groups averagely. On the seventh and eighth day after birth, each individual suckling mouse was challenged with two doses of 10^4.0^ CCID_50_ RV in 50μl via the oral route during a 24-hour period. Three standard RV strains (Wa, SA11 and Gottfried) were used. The faeces of the suckling mice were collected in PBS during the following 7 days and fecal samples were obtained via centrifugation at 3,000 rpm for 30 min and preserved at -80°C. ELISA tests were applied to analyze the RV shedding.

### Neutralization test

The neutralization activity of the immunized ICR mouse serum was measured against different genotypes RVs using a in vitro microneutralization assay, which was analyzed by observing CPEs. The 96-well plates were covered with a confluent monolayer of MA104 cells. The infectious titers of different genotypes activated RV strains were adjusted to 100 CCID_50_/100μl in serum-free MEM according to the infectious titers reported below. The immunized ICR mouse serum samples were diluted in serum-free MEM to a starting dilution of 1:20 in 100μl, and a series of seven additional 2-fold dilution steps was performed to generate a dilution range from 1:20 to 1:2560. The diluted RVs and serum samples were mixed with each other in another 96-well plate for 2 h at 37°C. Then, the mixtures were transferred to the 96-well plate covered with a confluent monolayer of MA104 cells and were cultured for 4–7 days at 37°C. The wells covered with MA104 cells in which there was just the 100 CCID_50_/ml of different genotypes RVs or the serum-free MEM were used as virus control or cell control. The results were observed under an optical microscope. A serum specimen was determined to be positive if no CPEs were observed in the MA104 cells in the well. The neutralization titers were defined as the reciprocal of the highest dilution.

### ELISA test for RV shedding

The wells of the ELISA plate were coated with a goat anti-rotavirus polyclonal antibody (Millipore, AB1129) diluted to 1:1000 (v/v) in carbonate-bicarbonate buffer (pH 9.6) overnight at 4°C. After washing three times with PBS containing 0.05% (v/v) Tween-20 (PBS-T), the ELISA plates were blocked with 2% (w/v) BSA (Biosharp, BS043D) diluted in PBS for 2 h at 37°C. Then, the fecal samples (100 μl/well) were incubated for 1 h at 37°C. The serum containing RV-specific antibodies (50 μl/well, diluted to 1:500 (v/v) in PBS) from guinea pig were incubated for 1 h at 37°C. After washing, the fecal samples were incubated in horseradish peroxidase (HRP)-conjugated goat anti-guinea pig IgG (H+L) (Millipore, AP108P) diluted to 1:2000 (v/v) in PBS for 1 h at 37°C. Soluble TMB Substrate Solution (TIANGEN, PA107-01) was used to generate a colorimetric reaction, which was terminated via the addition of 2 mol/L H_2_SO_4_. The optical densities (ODs) at 450 nm were measured using a universal microplate reader (ELx 800, Bio-Tek, USA). The PBS was used as negative controls. A fecal sample was determined to be positive if the OD value was greater than or equal to the average OD value of the negative control + 0.1.

### Statistical analysis

Data for neutralization test were expressed as GM*GSE^±tα/2,ν^. GM stood for Geometric Mean. GSE stood for Geometric Standard Error. In this study, α = 0.05 and ν = n-1, so the corresponding t value was able to be found according to the tables for statistical distributions (t-distribution) and the results reflected the 95% confidence interval. Comparisons between the neutralization titers in different groups and at different time-points were evaluated using Independent-Samples T Test. Comparisons between the percentages of RV shedding after challenge in different groups and at different time-points were evaluated using Fisher’s exact test. Statistical analyses were computed with SPSS 13.0. For all the analyses performed, P<0.05 was considered statistically significant.

## Results

### Measurement of the CCID_50_ of the RV immunogens

We first determined the infectious titers of purified RV suspensions. The calculated infectious titers of the different genotypes RV immunogens are listed in Table 1.

**Table 1:** Infectious titers of different genotypes RV immunogens.

RV strain	Genotype	Infectious titer (CCID_50_/ml) before purification	Infectious titer (CCID_50_/ml) after purification
Wa	G1P[8]	10^7.63^	10^7.40^
SA11	G3P[1]	10^7.83^	10^7.63^
Gottfried	G4P[6]	10^7.63^	10^7.56^

### Immunization of the experimental animals

The quantities of all the three RV immunogens were adjusted to 10^7.0^ CCID_50_/ml in PBS according to the infectious titers reported above. All the ICR mice used in this experiment had not been infected with other RVs via ELISA tests (shown in **Table in** S1 Table). The three genotypes of purified RV suspensions were mixed into different combinations at various proportions to generate multivalent RV immunogens. Then, ICR mice were immunized with the prepared monovalent and multivalent RV immunogens. The specific groups, sample sizes, infection routes and infection doses used in the animal experiments are listed in Table 2. No accidental death occurred, and all of the immunized ICR mice survived normally until the completion of the animal experiments.

**Table 2:** Groups, sample sizes, infection routes and infection doses in animal experiments.

Groups	Sample Sizes	Infection Routes	Infection Doses (CCID_50_/100μl)
Wa	8	oral route	10^6.0^
SA11	8	oral route	10^6.0^
Gottfried	8	oral route	10^6.0^
Wa+SA11	8	oral route	1/2*10^6.0^+1/2*10^6.0^
Wa+Gottfried	8	oral route	1/2*10^6.0^+1/2*10^6.0^
SA11+Gottfried	8	oral route	1/2*10^6.0^+1/2*10^6.0^
Wa+SA11+Gottfried	8	oral route	1/3*10^6.0^+1/3*10^6.0^+1/3*10^6.0^
PBS	8	oral route	0

### ELISA test for serum RV-specific antibodies

**Fig 1:**
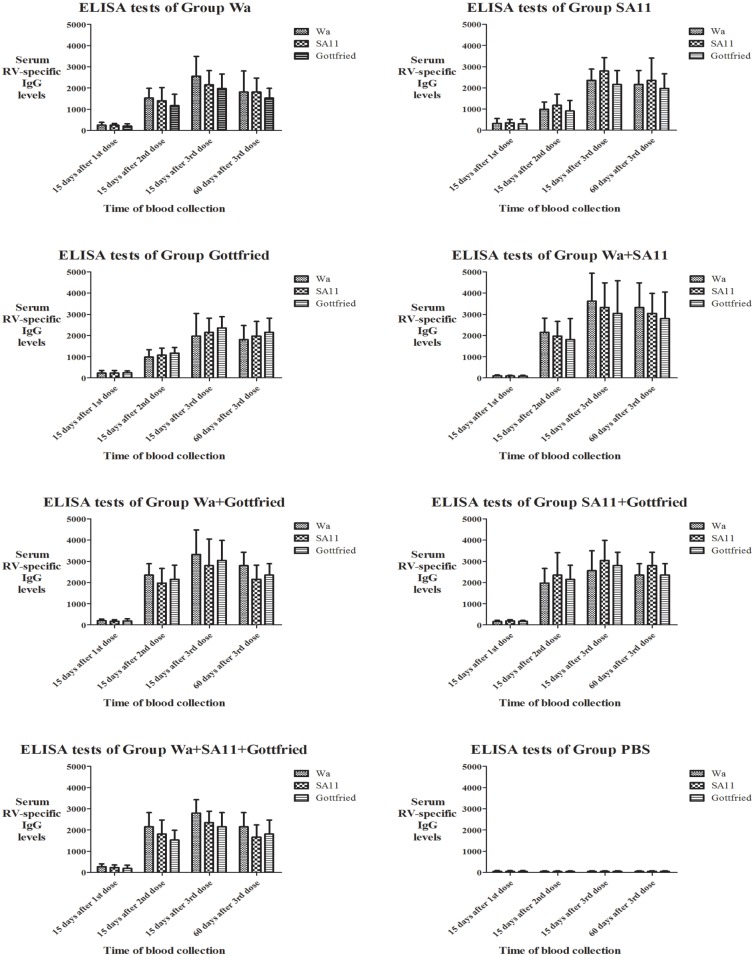
ELISA test results of every group. The serum RV-specific IgG levels of each serum sample in every group were detected by ELISA tests against all the three RV immunogens Wa (G1P[8] genotype), SA11 (G3P[1] genotype) and Gottfried (G4P[6] genotype) after every dose. Data were expressed as GM*GSE^±tα/2,ν^. GM stood for Geometric Mean. GSE stood for Geometric Standard Error. In this study, α = 0.05 and ν = n-1 = 8–1 = 7, so the corresponding t value was able to be found according to the tables for statistical distributions (t-distribution). The heights of columns were based on the GM of each test and the error bar stood for the 95% confidence interval.

The results of the ELISA test are shown in Fig 1 and **Table in** S2 Table. According to the results, both the monovalent and multivalent RV immunogens revealed high immunogenicity and induced the production of serum RV-specific IgG in the immunized ICR mice compared with injection with PBS (P<0.05). Booster immunization with the 2rd and 3nd dose could dramatically enhance the production of serum RV-specific IgG (P<0.05).

### Neutralization test

**Fig 2:**
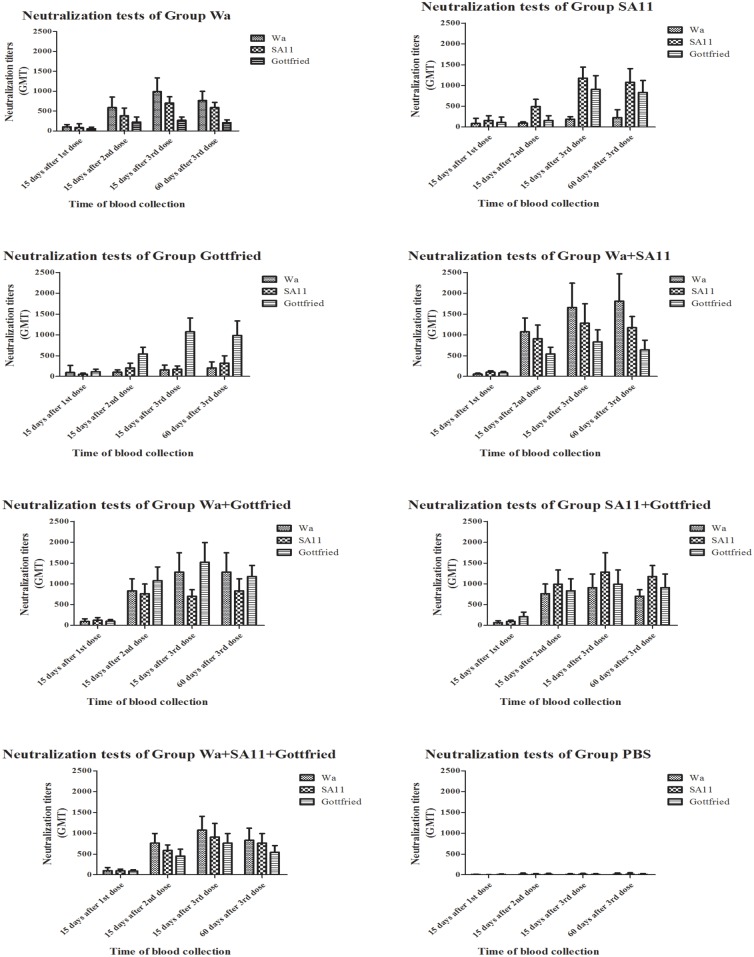
Neutralization test results of every group. The neutralization titers of each serum sample in every group were detected by neutralization tests against all the three RV immunogens Wa (G1P[8] genotype), SA11 (G3P[1] genotype) and Gottfried (G4P[6] genotype) after every dose. Data were expressed as GM*GSE^±tα/2,ν^. GM stood for Geometric Mean. GSE stood for Geometric Standard Error. In this study, α = 0.05 and ν = n-1 = 8–1 = 7, so the corresponding t value was able to be found according to the tables for statistical distributions (t-distribution). The heights of columns were based on the GM of each test and the error bar stood for the 95% confidence interval.

The results of the neutralization test are shown in Fig 2 and **Table in** S3 Table. According to the results, both the monovalent and multivalent RV immunogens exhibited high immunogenicity and stimulated the production of RV-specific neutralization antibodies in the immunized ICR mice compared with injection with PBS (P<0.05). Booster immunization with the 2rd and 3nd dose could evidently enhance the production of RV-specific neutralization antibodies (P<0.05). Concerning the maintenance of immunity, the neutralization titers of all of the groups generally decreased 60 days after the 3rd dose (P<0.05), indicating that the maintenance of neutralization antibodies might require additional booster immunizations if necessary.

As shown in the above results and in the results of previous studies, RV immunogens were generally immunogenic. Both monovalent and multivalent RV immunogens could provoke the production of RV-specific neutralization antibodies. However, a significant difference was detected between the monovalent and multivalent RV immunogens. The ICR mice were immunized with the same total RV immunogen quantity. Under this circumstance, the results revealed that the neutralization titers in response to stimulation with the multivalent RV immunogens (Group Wa+SA11, Group Wa+Gottfried, Group SA11+Gottfried and Group Wa+SA11+Gottfried) were higher than those in response to stimulation with the monovalent RV immunogens (Group Wa, Group SA11 and Group Gottfried) (P<0.05).

**Fig 3:**
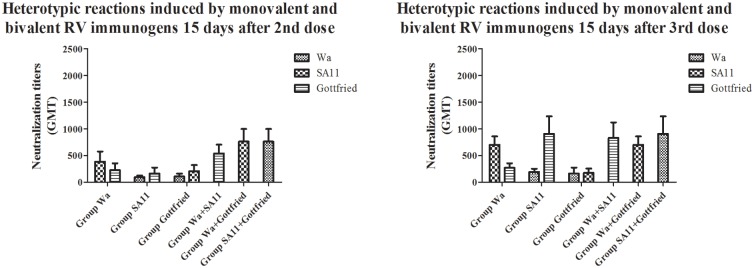
Comparative analysis of heterotypic reactions between monovalent and bivalent RV immunogens. The heterotypic reactions were evaluated by comparing the neutralization titers against heterotypic RV strains that were not contained in monovalent and bivalent RV immunogens 15 days after 2nd and 3rd dose.

Heterotypic reaction elicited by RVs is a character of RV immunogens because of their diversity [20]. When it came to the heterotypic reaction, the RV-specific neutralization antibodies elicited by monovalent and multivalent RV immunogens exhibited different efficacities on the fighting against infection with heterotypic RV strains carrying different G and P genotypes. We clearly observed that both the monovalent and multivalent RV immunogens displayed heterotypic reactions. But all the multivalent RV immunogens induced high-level heterotypic reactions 15 days after 2nd dose, whereas the most monovalent RV immunogens often induced high-level heterotypic reactions 15 days after 3rd dose and the heterotypic reaction elicited by the monovalent RV immunogen Group Gottfried remained a lower level even 15 days after 3rd dose (shown in Fig 3), which suggested that the multivalent RV immunogens could induce heterotypic reactions more rapidly and the monovalent RV immunogens require additional inoculations for effective heterotypic reactions to induce heterotypic neutralization antibodies against diverse RV infections.

**Fig 4:**
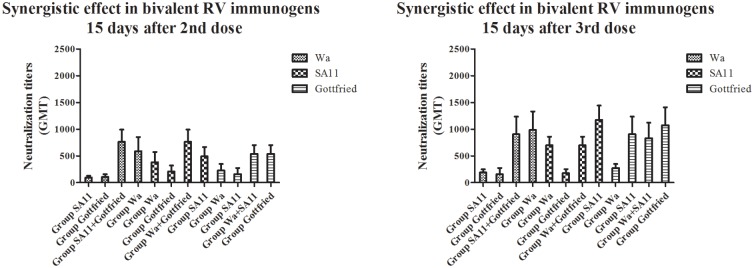
Synergistic effect in bivalent RV immunogens. The heterotypic reactions induced by bivalent RV immunogens were stronger than those induced by monovalent RV immunogens which were the components of bivalent RV immunogens (P<0.05). And the heterotypic reactions induced by bivalent RV immunogens were even at the same level as the homotypic reactions induced by the homotypic monovalent RV immunogens (P>0.05).

Why could multivalent RV immunogens induce more rapid and stronger heterotypic reactions than monovalent RV immunogens under the circumstance that the ICR mice were immunized with the same total RV immunogen quantity? We further compared the neutralization titers between two monovalent RV immunogens and the combination of them (bivalent RV immunogen) against another heterotypic RV strain and found that the synergistic effect between different RV strains contained in the multivalent RV immunogens might be the cornerstone of the high-level heterotypic reaction (shown in Fig 4). For example, the Group SA11 and Group Gottfried displayed low neutralization titers against Wa, but the Group SA11+Gottfried displayed remarkably higher neutralization titers against Wa (P<0.05) 15 days after 2nd and 3rd dose. Moreover, no significant difference was detected in the neutralization titers against Wa between the Group SA11+Gottfried and Group Wa 15 days after 2nd and 3rd dose (P>0.05), implying that the heterotypic reaction caused by the multivalent RV immunogens could provide enough neutralization antibodies to protect MA104 cells against the infection with RVs as the homotypic reaction caused by the monovalent RV immunogens although it is understood that homotypic reaction is stronger than heterotypic reaction. Similar results happened to the comparison between other monovalent and multivalent RV immunongens (shown in Fig 4). The monovalent RV immunogens could not represent heterotypic reactions against some heterotypic RV strains even 15 days after 3rd dose.

**Fig 5:**
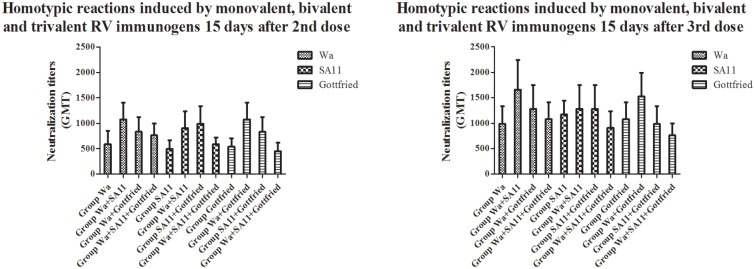
Comparative analysis of homotypic reactions between monovalent, bivalent and trivalent RV immunogens. The homotypic reactions were evaluated by comparing the neutralization titers against homootypic RV strains that were contained in monovalent, bivalent and trivalent RV immunogens 15 days after 2nd and 3rd dose.

Another interesting phenomenon was observed in the comparison between the monovalent, bivalent and trivalent RV immunogens, it was found that the neutralization titers against homotypic RV strains of the bivalent RV immunogens were generally higher than those of the monovalent and trivalent RV immunogens (P<0.05) (shown in Fig 5). For example, the Group Wa+SA11 and Group Wa+Gottfried induced higher neutralization titers against Wa than the Group Wa (P<0.05), because of the synergistic effect between different RV strains which we have mentioned above. The Group Wa+SA11 and Group Wa+Gottfried also induced higher neutralization titers against Wa than the Group Wa+SA11+Gottfried, even though all the three groups were infected with multivalent RV immunogens containing Wa (P<0.05). Under the condition that each group was treated with the same total RV immunogen quantity, it was speculated that the proportion of the RV strain that induced a homotypic reaction played an important role in the production of neutralization antibodies. The proportions of Wa were different between the bivalent (1/2) and trivalent RV immunogens (1/3), which might be the reason for the different neutralization titers against Wa between infection with the bivalent and trivalent RV immunogens. Therefore, it was not necessarily true that the more RV genotypes that the multivalent RV immunogens contained, the higher the neutralization titer would be. The quantity of a single RV strain and the categories of genotypes of all RV strains in the multivalent RV immunogens were of equal importance.

### Efficacy against RV challenge

**Fig 6:**
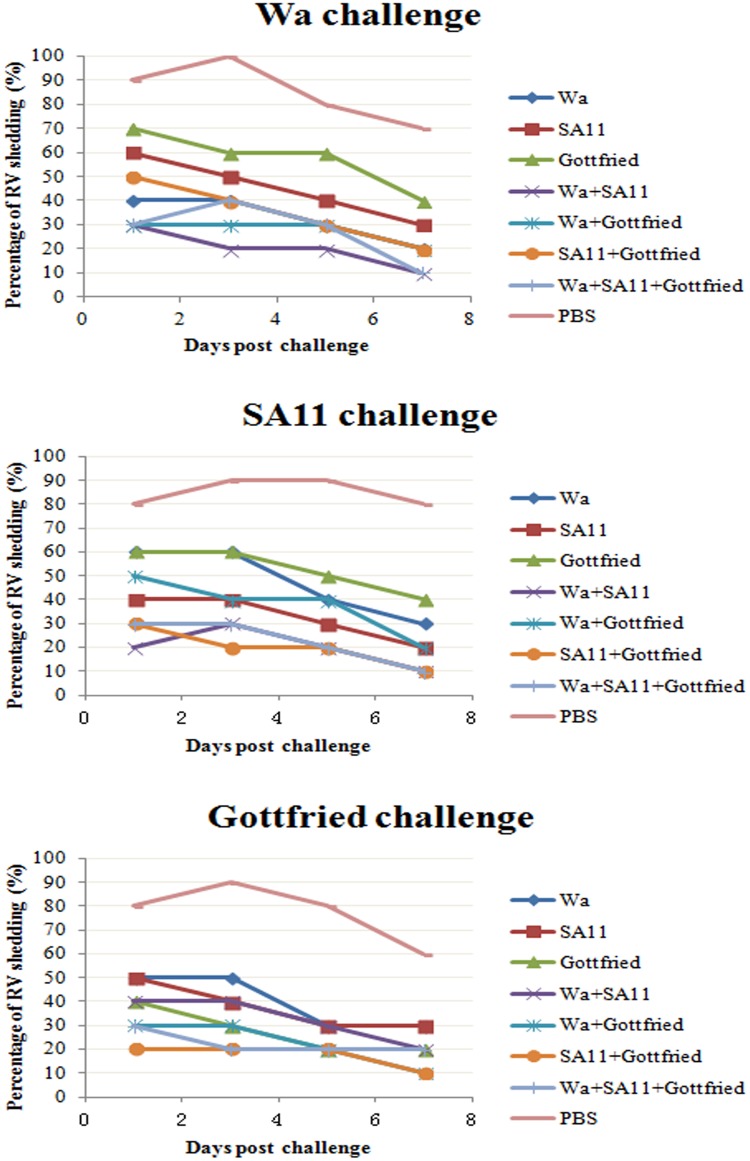
Percentage of RV fecal shedding in suckling mice after RV strains challenge. Suckling mice have required passive immunity from their mother mice. Fecal samples were collected from suckling mice, processed and RV-antigen detected by ELISA test after RV strains challenge. Virus infection rates were expressed as percentage of RV shedding.

Efficacy was measured as the percent reduction in RV shedding, comparing the antigen shed per suckling mouse per day in the passive immunized groups (monovalent and multivalent RV immunogens groups) to the control group (PBS group) during days 1–7 following RV strains challenge (shown in Fig 6 and **Table in** S4 Table).

According to the results, the reduction in shedding percent was observed in both monovalent and multivalent RV immunogens immunization groups during 7 days after diverse RV strains challenge in comparison with the PBS group (P<0.05), which suggested that both the monovalent and multivalent RV immunogens could provide the protection against homotypic and heterotypic RV strains’ infection. However, a significant difference was observed between the monovalent and multivalent RV immunogens especially on the foremost days after RV strains challenge (P<0.05). As time went on (7th day after challenge), the percentage of RV shedding in both monovalent and multivalent RV immunogens groups would be at the same level (P>0.05). But on the foremost days (1st and 3rd day) after challenge, the percentage of RV shedding in the monovalent RV immunogen groups were higher than those in the multivalent RV immunogen groups (P<0.05), which suggested that the multivalent RV immunogens could induce the protection more rapidly than the monovalent RV immunogens.

## Discussion

To date, both monovalent and multivalent vaccines have been primary strategies of RV vaccine development. However, more and more researches and clinical trials have demonstrated that monovalent RV vaccine could provide effective protection against homotypic and heterotypic RV strains [21–22]. What is more, the preparation technology of monovalent RV vaccine is simpler and monovalent RV vaccine is more cost-effective than multivalent RV vaccine. Therefore, monovalent RV vaccine is more preferred by researchers in the development of RV vaccine. Many of the RV vaccines in clinical trials are monovalent RV vaccines, such as 116E RV vaccine (G9P[11]), Rotavin-M1 RV vaccine (G1P[8]) and RV3-BB RV vaccine (G3P[6]) [23–25]. According to the results in this study, both the monovalent and multivalent RV immunogens displayed high immunogenicity, but the monovalent RV immunogens could not provide enough neutralization antibodies to protect MA104 cells against the infection with heterotypic RV strains while the synergistic effect between different RV strains contained in the multivalent RV immunogens might promote more rapid and stronger heterotypic reaction which provided more effective serum neutralization antibodies. As for the protection, the results demonstrated that the multivalent RV immunogens could induce the protection against homotypic and heterotypic RV strains’ infection more rapidly than the monovalent RV immunogens, which was similar to the results of the neutralization test. In addition, several reports have suggested that the heterotypic protection elicited by monovalent RV vaccine could not protect against all the heterotypic RV strains and parts of the results corresponded to this conclusion [17, 21]. Therefore, as the diversity of circulating RV strains increasing, multivalent vaccines are necessary for the future development of RV vaccine and are not able to be replaced by monovalent vaccines. Which type of RV vaccine (monvalent or multivalent RV vaccine) should be the direction of the development of RV vaccine in the future needs a comprehensive consideration. However, several issues remain to be resolved before the development of novel RV vaccines, and the most crucial issue is determining the mechanism of heterotypic reaction and cross protection. The new field of science referred to as systems biology, which has been applied to yellow fever and influenza vaccines, may be adopted to elucidate the mechanism underlying the immunity of organisms after RV infection [26–28]. This information would be helpful to determine the combination of RVs that induce optimal heterotypic reaction and cross protection, which could provide a theoretical basis for the future development of multivalent RV vaccines.

## Supporting information

S1 Table ELISA tests for serum RV-specific IgG levels before immunization. (DOCX)

S2 Table ELISA tests for serum RV-specific IgG levels. (DOCX)

S3 Table Neutralization test for neutralization titers. (DOCX)

S4 Table Percentage of RV fecal shedding in suckling mice after RV strains challenge. (DOCX)
