# Supplementary material for: Comparative analysis of the immunogenicity of monovalent and multivalent rotavirus immunogens
Source: PLoS One. 2017 Feb 16;12(2):e0172156. doi: 10.1371/journal.pone.0172156 (PMC5313208; doi:10.1371/journal.pone.0172156)
Supplement: S1 Table — (DOCX) [file pone.0172156.s001.docx]

**ELISA tests for serum RV-specific IgG levels before immunization**

| **Groups** | **Sample Sizes** | **Serum RV-specific IgG levels** | | | | | | | |
| --- | --- | --- | --- | --- | --- | --- | --- | --- | --- |
| **Wa** | 8 | 1 | 10 | 1 | 1 | 10 | 1 | 10 | 1 |
| **SA11** | 8 | 1 | 1 | 1 | 1 | 1 | 1 | 1 | 1 |
| **Gottfried** | 8 | 1 | 10 | 10 | 1 | 1 | 1 | 1 | 1 |
| **Wa+SA11** | 8 | 1 | 1 | 1 | 1 | 1 | 1 | 1 | 1 |
| **Wa+Gottfried** | 8 | 10 | 1 | 1 | 10 | 1 | 1 | 1 | 1 |
| **SA11+Gottfried** | 8 | 1 | 1 | 1 | 1 | 1 | 10 | 1 | 1 |
| **Wa+SA11+Gottfried** | 8 | 1 | 1 | 10 | 1 | 1 | 10 | 1 | 1 |
| **PBS** | 8 | 1 | 1 | 1 | 1 | 10 | 1 | 1 | 1 |

Prior to immunization, the serums of all the ICR mice used in this experiment were detected via ELISAs. The serum RV-specific IgG levels were defined as the reciprocal of the highest dilution. The results of ELISAs showed that the serum RV-specific IgG levels of all the ICR mice were less than 10, which suggested that the ICR mice had not been infected with other RVs.
