# Supplementary material for: Comparative analysis of the immunogenicity of monovalent and multivalent rotavirus immunogens
Source: PLoS One. 2017 Feb 16;12(2):e0172156. doi: 10.1371/journal.pone.0172156 (PMC5313208; doi:10.1371/journal.pone.0172156)
Supplement: S2 Table — (DOCX) [file pone.0172156.s002.docx]

**ELISA tests for serum RV-specific IgG levels**

| Group | Antigen | 15 days after  1st dose | 15 days after  2nd dose | 15 days after  3rd dose | 60 days after  3rd dose |
| --- | --- | --- | --- | --- | --- |
| Wa | Wa | 246.75*1.20^±2.365^ | 1522.19*1.12^±2.365^ | 2560.00*1.14^±2.365^ | 1810.19*1.20^±2.365^ |
|  | SA11 | 246.75*1.14^±2.365^ | 1395.85*1.17^±2.365^ | 2152.69*1.12^±2.365^ | 1810.19*1.14^±2.365^ |
|  | Gottfried | 207.49*1.20^±2.365^ | 1173.77*1.17^±2.365^ | 1974.03*1.14^±2.365^ | 1522.19*1.12^±2.365^ |
| SA11 | Wa | 320.00*1.25^±2.365^ | 987.01*1.14^±2.365^ | 2347.53*1.09^±2.365^ | 2152.69*1.12^±2.365^ |
|  | SA11 | 348.96*1.17^±2.365^ | 1173.77*1.17^±2.365^ | 2791.70*1.09^±2.365^ | 2347.53*1.17^±2.365^ |
|  | Gottfried | 293.44*1.27^±2.365^ | 905.10*1.20^±2.365^ | 2152.69*1.12^±2.365^ | 1974.03*1.14^±2.365^ |
| Gottfried | Wa | 226.27*1.20^±2.365^ | 987.01*1.14^±2.365^ | 1974.03*1.20^±2.365^ | 1810.19*1.14^±2.365^ |
|  | SA11 | 226.27*1.20^±2.365^ | 1076.35*1.12^±2.365^ | 2152.69*1.12^±2.365^ | 1974.03*1.14^±2.365^ |
|  | Gottfried | 246.75*1.14^±2.365^ | 1173.77*1.09^±2.365^ | 2347.53*1.09^±2.365^ | 2152.69*1.12^±2.365^ |
| Wa+  SA11 | Wa | 103.75*1.14^±2.365^ | 2152.69*1.12^±2.365^ | 3620.39*1.14^±2.365^ | 3319.91*1.14^±2.365^ |
|  | SA11 | 95.14*1.12^±2.365^ | 1974.03*1.14^±2.365^ | 3319.91*1.14^±2.365^ | 3044.37*1.12^±2.365^ |
|  | Gottfried | 87.24*1.17^±2.365^ | 1810.19*1.20^±2.365^ | 3044.37*1.19^±2.365^ | 2791.70*1.17^±2.365^ |
| Wa+  Gottfried | Wa | 207.49*1.14^±2.365^ | 2347.53*1.09^±2.365^ | 3319.91*1.14^±2.365^ | 2791.70*1.09^±2.365^ |
|  | SA11 | 174.48*1.17^±2.365^ | 1974.03*1.14^±2.365^ | 2791.70*1.17^±2.365^ | 2152.69*1.12^±2.365^ |
|  | Gottfried | 190.27*1.19^±2.365^ | 2152.69*1.12^±2.365^ | 3044.37*1.12^±2.365^ | 2347.53*1.09^±2.365^ |
| SA11+  Gottfried | Wa | 160.00*1.14^±2.365^ | 1974.03*1.14^±2.365^ | 2560.00*1.14^±2.365^ | 2347.53*1.09^±2.365^ |
|  | SA11 | 190.27*1.12^±2.365^ | 2347.53*1.17^±2.365^ | 3044.37*1.12^±2.365^ | 2791.70*1.09^±2.365^ |
|  | Gottfried | 174.48*1.09^±2.365^ | 2152.69*1.12^±2.365^ | 2791.70*1.09^±2.365^ | 2347.53*1.09^±2.365^ |
| Wa+  SA11+  Gottfried | Wa | 269.09*1.19^±2.365^ | 2152.69*1.12^±2.365^ | 2791.70*1.09^±2.365^ | 2152.69*1.12^±2.365^ |
|  | SA11 | 226.27*1.20^±2.365^ | 1810.19*1.14^±2.365^ | 2347.53*1.09^±2.365^ | 1659.95*1.14^±2.365^ |
|  | Gottfried | 190.27*1.29^±2.365^ | 1522.19*1.12^±2.365^ | 2152.69*1.12^±2.365^ | 1810.19*1.14^±2.365^ |
| PBS | Wa | 51.87*1.25^±2.365^ | 43.62*1.23^±2.365^ | 47.57*1.24^±2.365^ | 47.57*1.24^±2.365^ |
|  | SA11 | 51.87*1.25^±2.365^ | 43.62*1.23^±2.365^ | 47.57*1.24^±2.365^ | 47.57*1.24^±2.365^ |
|  | Gottfried | 51.87*1.25^±2.365^ | 43.62*1.23^±2.365^ | 47.57*1.24^±2.365^ | 47.57*1.24^±2.365^ |

The data were expressed as GM*GSE^±2.365^ which reflected the 95% confidence interval.

GM: Geometric Mean; GSE: Geometric Standard Error; and t_α/2_,_ν_: in this study, α=0.05, ν=n-1=8-1=7, t=2.365 (refer to tables for statistical t-distribution).
