# Supplementary material for: Comparative analysis of the immunogenicity of monovalent and multivalent rotavirus immunogens
Source: PLoS One. 2017 Feb 16;12(2):e0172156. doi: 10.1371/journal.pone.0172156 (PMC5313208; doi:10.1371/journal.pone.0172156)
Supplement: S3 Table — (DOCX) [file pone.0172156.s003.docx]

**Neutralization test for neutralization titers**

| Group | Antigen | 15 days after  1st dose | 15 days after  2nd dose | 15 days after  3rd dose | 60 days after  3rd dose |
| --- | --- | --- | --- | --- | --- |
| Wa | Wa | 103.75*1.20^±2.365^ | 586.88*1.17^±2.365^ | 987.01*1.14^±2.365^ | 761.09*1.12^±2.365^ |
|  | SA11 | 87.24*1.36^±2.365^ | 380.55*1.19^±2.365^ | 697.92*1.09^±2.365^ | 586.88*1.09^±2.365^ |
|  | Gottfried | 56.57*1.25^±2.365^ | 226.27*1.20^±2.365^ | 269.09*1.12^±2.365^ | 207.49*1.14^±2.365^ |
| SA11 | Wa | 87.24*1.46^±2.365^ | 95.14*1.12^±2.365^ | 190.27*1.12^±2.365^ | 226.27*1.30^±2.365^ |
|  | SA11 | 160.00*1.25^±2.365^ | 493.51*1.14^±2.365^ | 1173.77*1.09^±2.365^ | 1076.35*1.12^±2.365^ |
|  | Gottfried | 113.14*1.38^±2.365^ | 160.00*1.25^±2.365^ | 905.10*1.14^±2.365^ | 829.98*1.14^±2.365^ |
| Gottfried | Wa | 95.14*1.54^±2.365^ | 103.75*1.20^±2.365^ | 160.00*1.25^±2.365^ | 207.49*1.25^±2.365^ |
|  | SA11 | 51.87*1.20^±2.365^ | 207.49*1.20^±2.365^ | 174.48*1.17^±2.365^ | 320.00*1.20^±2.365^ |
|  | Gottfried | 113.14*1.20^±2.365^ | 538.17*1.12^±2.365^ | 1076.35*1.12^±2.365^ | 987.01*1.14^±2.365^ |
| Wa+  SA11 | Wa | 56.57*1.14^±2.365^ | 1076.35*1.12^±2.365^ | 1659.95*1.14^±2.365^ | 1810.19*1.14^±2.365^ |
|  | SA11 | 103.75*1.14^±2.365^ | 905.10*1.14^±2.365^ | 1280.00*1.14^±2.365^ | 1173.77*1.09^±2.365^ |
|  | Gottfried | 87.24*1.17^±2.365^ | 538.17*1.12^±2.365^ | 829.98*1.14^±2.365^ | 640.00*1.14^±2.365^ |
| Wa+  Gottfried | Wa | 95.14*1.24^±2.365^ | 829.98*1.14^±2.365^ | 1280.00*1.14^±2.365^ | 1280.00*1.14^±2.365^ |
|  | SA11 | 123.38*1.20^±2.365^ | 761.09*1.12^±2.365^ | 697.92*1.09^±2.365^ | 829.98*1.14^±2.365^ |
|  | Gottfried | 103.75*1.14^±2.365^ | 1076.35*1.12^±2.365^ | 1522.19*1.12^±2.365^ | 1173.77*1.09^±2.365^ |
| SA11+  Gottfried | Wa | 67.27*1.24^±2.365^ | 761.09*1.12^±2.365^ | 905.10*1.14^±2.365^ | 697.92*1.09^±2.365^ |
|  | SA11 | 95.14*1.12^±2.365^ | 987.01*1.14^±2.365^ | 1280.00*1.14^±2.365^ | 1173.77*1.09^±2.365^ |
|  | Gottfried | 207.49*1.20^±2.365^ | 829.98*1.14^±2.365^ | 987.01*1.14^±2.365^ | 905.10*1.14^±2.365^ |
| Wa+  SA11+  Gottfried | Wa | 103.75*1.25^±2.365^ | 761.09*1.12^±2.365^ | 1076.35*1.12^±2.365^ | 829.98*1.14^±2.365^ |
|  | SA11 | 103.75*1.14^±2.365^ | 586.88*1.09^±2.365^ | 905.10*1.14^±2.365^ | 761.09*1.12^±2.365^ |
|  | Gottfried | 95.14*1.12^±2.365^ | 452.55*1.14^±2.365^ | 761.09*1.12^±2.365^ | 538.17*1.12^±2.365^ |
| PBS | Wa | 3.35*1.81^±2.365^ | 8.43*1.88^±2.365^ | 5.32*1.89^±2.365^ | 9.20*1.95^±2.365^ |
|  | SA11 | 1.59*1.59^±2.365^ | 7.09*1.79^±2.365^ | 10.31*1.68^±2.365^ | 10.03*1.96^±2.365^ |
|  | Gottfried | 4.47*1.76^±2.365^ | 6.32*2.01^±2.365^ | 6.50*1.73^±2.365^ | 7.09*1.79^±2.365^ |

The data were expressed as GM*GSE^±2.365^ which reflected the 95% confidence interval.

GM: Geometric Mean; GSE: Geometric Standard Error; and t_α/2_,_ν_: in this study, α=0.05, ν=n-1=8-1=7, t=2.365 (refer to tables for statistical t-distribution).
