# Supplementary material for: Comparative analysis of the immunogenicity of monovalent and multivalent rotavirus immunogens
Source: PLoS One. 2017 Feb 16;12(2):e0172156. doi: 10.1371/journal.pone.0172156 (PMC5313208; doi:10.1371/journal.pone.0172156)
Supplement: S4 Table — (DOCX) [file pone.0172156.s004.docx]

**Percentage of RV fecal shedding in suckling mice after RV strains challenge**

| RVs for challenge | Groups | Sample sizes | Percentage of RV shedding (%) | | | |
| --- | --- | --- | --- | --- | --- | --- |
|  |  |  | DPC-1 | DPC-3 | DPC-5 | DPC-7 |
| Wa | Wa | 10 | 40 | 40 | 30 | 20 |
|  | SA11 | 10 | 60 | 50 | 40 | 30 |
|  | Gottfried | 10 | 70 | 60 | 60 | 40 |
|  | Wa+SA11 | 10 | 30 | 20 | 20 | 10 |
|  | Wa+Gottfried | 10 | 30 | 30 | 30 | 20 |
|  | SA11+Gottfried | 10 | 50 | 40 | 30 | 20 |
|  | Wa+SA11+Gottfried | 10 | 30 | 40 | 30 | 10 |
|  | PBS | 10 | 90 | 100 | 80 | 70 |
| SA11 | Wa | 10 | 60 | 60 | 40 | 30 |
|  | SA11 | 10 | 40 | 40 | 30 | 20 |
|  | Gottfried | 10 | 60 | 60 | 50 | 40 |
|  | Wa+SA11 | 10 | 20 | 30 | 20 | 10 |
|  | Wa+Gottfried | 10 | 50 | 40 | 40 | 20 |
|  | SA11+Gottfried | 10 | 30 | 20 | 20 | 10 |
|  | Wa+SA11+Gottfried | 10 | 30 | 30 | 20 | 10 |
|  | PBS | 10 | 80 | 90 | 90 | 80 |
| Gottfried | Wa | 10 | 50 | 50 | 30 | 30 |
|  | SA11 | 10 | 50 | 40 | 30 | 30 |
|  | Gottfried | 10 | 40 | 30 | 20 | 20 |
|  | Wa+SA11 | 10 | 40 | 40 | 30 | 20 |
|  | Wa+Gottfried | 10 | 30 | 30 | 20 | 10 |
|  | SA11+Gottfried | 10 | 20 | 20 | 20 | 10 |
|  | Wa+SA11+Gottfried | 10 | 30 | 20 | 20 | 20 |
|  | PBS | 10 | 80 | 90 | 80 | 60 |

DPC: days post challenge
